# Lessons from Israel's COVID-19 Green Pass program

**DOI:** 10.1186/s13584-021-00496-4

**Published:** 2021-10-29

**Authors:** Shelly Kamin-Friedman, Maya Peled Raz

**Affiliations:** 1 Department of Health Policy and Management, School of Public Health, Faculty of Health Sciences, Ben-Gurion University of the Negev, Be’er Sheva, Israel; 2 Faculty of Law, Haifa University, Haifa, Israel; 3 The School of Public Health, The Center for Health, Law and Ethics, University of Haifa, Haifa, Israel; 4 Clinical Ethicist, Ethics Committee Chair, Bnai Zion Medical Center, Haifa, Israel

**Keywords:** Green pass, Covid-19, Vaccination, Vaccine, Fairness, Solidarity, Public health ethics

## Abstract

As of the beginning of March 2021, Israeli law requires the presentation of a Green Pass as a precondition for entering certain businesses and public spheres. Entitlement for a Green Pass is granted to Israelis who have been vaccinated with two doses of COVID-19 vaccine, who have recovered from COVID-19, or who are participating in a clinical trial for vaccine development in Israel. The Green Pass is essential for retaining immune individuals' freedom of movement and for promoting the public interest in reopening the economic, educational, and cultural spheres of activity. Nonetheless, and as the Green Pass imposes restrictions on the movement of individuals who had not been vaccinated or who had not recovered, it is not consonant with solidarity and trust building. Implementing the Green Pass provision while advancing its effectiveness on the one hand, and safeguarding equality, proportionality, and fairness on the other hand may imbue this measure with ethical legitimacy despite involving a potential breach of trust and solidarity.

## Key points

- Although the Green Pass may not be consonant with trust building or with the promotion of solidarity, it is essential for the realization of the freedom of movement and gathering and for resuming social activity.
- The possession of a Green Pass as a pre-condition for entering businesses and public places would only be an effective measure for reopening the economy and the cultural sphere of activity if it generates an economic incentive to businesses and venue owners.
- The implementation of the Green Pass program underscores the need for making vaccines accessible to vulnerable, and especially poor populations.
- Requiring employees to present a Green Pass as a condition of admission to their workplaces is proportionate if employees are given the alternative of undergoing PCR or rapid antigen tests, and if the presentation of a Green Pass is essential for safe activity.

## Introduction

As of the beginning of March 2021, Israelis who have been vaccinated with two doses of COVID-19 vaccine, who have recovered from COVID-19, or who are currently participating in a clinical trial for vaccine development in Israel are entitled to a Green Pass. The Green Pass is a certificate which allows its holder to establish her or his immunization status and take part in various activities such as cultural and sports events, conferences, gym classes and workouts, exhibitions, swimming in public swimming pools, hotel stays, eating out at restaurants, and visiting tourist attractions, which were all closed intermittently since April 2020.

Provisions concerning the Green Pass are regulated by secondary legislation [1] enacted by the executive branch within its authority to act to reduce the spread of the virus [2]. The Green Pass may be legally required for a changeable variety of venues and activities depending on the severity of the pandemic as advised by experts.

In spite of the uncertainty surrounding the long-term effectiveness of the mRNA COVID-19 vaccine as well as its effectiveness against variants, the implementation of the Green Pass scheme was based on research confirming that infection with COVID-19 induces an immune response [3], and that vaccines are (as of now) effective in both preventing severe morbidity [4] and in reducing infection [5–8]. These assumptions hold in the face of the delta variant, though effectiveness in both aspects is estimated as lower compared to the alpha variant [9].

The ethics of the Green Pass can be assessed with respect to general moral considerations in public health, and first and foremost with respect to the aspiration toward the maximal balance of benefits over harms and other costs; the fair distribution of benefits and burdens; trust building, and promotion of solidarity [10–12].

The Green Pass allows immune individuals to retain their freedoms of movement and gathering, and promotes the public interest in reopening the economic, educational and cultural spheres of activity.

Nonetheless, and as the Green Pass program imposes restrictions on the movement of individuals who had not been vaccinated or who had not recovered, it is not consonant with the solidarity and trust building that are both central to public health.^1^

Unlike a call for a collective commitment to the common good without individual restrictions (as was utilized in Israel during the detection of poliovirus in 2013), the coercive legislation, which excludes unvaccinated individuals from businesses and public places can lead to a sense of alienation. Moreover, restrictions on individuals express distrust on the part of health authorities with respect to the non Green Pass holders' commitment to the collective efforts toward overcoming the pandemic.

The present article’s purpose is thus to examine the Israeli experience in engaging and balancing ethical values during the implementation of the Green Pass program.

## The necessity of the Green Pass

Vaccine uptake in Israel is extensive. As of October 15, 2021, 64.8% of the population was vaccinated with 2 vaccine doses, including a very high proportion of the at-risk population (84.6% aged 50–59; 88.3% aged 60–69; 88.1% aged 70–79; 87.7% aged 80–89; and 80.2% over the age of 90). Nonetheless, there are currently about 700,000 Israelis who are entitled to receive the vaccine but who have nonetheless chosen not to get vaccinated.

The Green Pass was introduced in Israel with a dual purpose in mind: to allow (following the third COVID-19 wave) and to maintain (in the wake of the fourth wave) the opening of the economy, the education system, and the cultural sphere with a minimum risk of elevating morbidity, as well as to encourage vaccination [14].

The opening of the economy, the education system, and the cultural sphere is essential to social resilience [15]. Moreover, allowing vaccinated or recovered individuals to resume and maintain their daily routine is consonant with their rights to freedom of movement and gathering. Individuals who are immune to SARS-CoV-2 are expected to be at a vastly reduced risk of contracting and transmitting the virus, and requiring them to keep complying with social distance restrictions infringes extensively on their civil liberties and would be unjustified. Consequently, it is unethical to require people to avoid contact with others if they pose a minimal risk of spreading the virus [16].^2^

Therefore, and although the Green Pass may not correlate with trust building or the promotion of solidarity, it is ethically vital to consider its application under the Israeli circumstances.

## The Green Pass as an effective measure for safely re-opening the economy

The assumption that the Green Pass is essential to the realization of the freedom of movement and gathering and to safely reopening the economy does not necessarily mean that it would be an effective measure under all circumstances.

Businesses and venue owners would only resume their activity following the Green Pass restrictions if they had an economic incentive for doing so.

This economic incentive would be diminished if the legislature applies further restrictions on the operation of the relevant businesses (i.e., limiting the number of customers allowed in or prohibiting the sale of food on the premises) over and above the requirement of barring non-Green Pass holders from entering the premises.

Moreover, an economic incentive hardly applies to businesses relying on a clientele consisting mainly of children under 12 who cannot presently be vaccinated and who do not possess a Green Pass. For example, the owners of Israeli amusement parks have stated that opening their business to Green Pass holders alone would not be profitable and hence not feasible [18].

Another relevant factor for the resumption of business activity under Green Pass provisions is the businesses’ responsibility for its enforcement. In Israel, the duty to ensure that all those who enter venues operating under the Green Pass restrictions do indeed possess a Green Pass rests with the businesses' owners. Allowing entry to a non-Green Pass holder is an administrative offense punishable by a fine of 5000–10,000 NIS (ca. 1550–3100 USD) payable by the business owner. Business owners have argued that enforcing the Green Pass provisions requires the hiring of additional employees and reduces the economic feasibility of the businesses’ opening.

Policy makers must therefore bear in mind that businesses will open in accordance with Green Pass provisions and allow vaccinated or recovered individuals to resume their routine if solutions are found for allowing children to enter relevant venues (for example, rapid COVID-19 testing as an alternative to the presentation of a Green Pass), and if other business restrictions or obligations are limited to a minimum.

It should also be borne in mind that businesses will only have an incentive to resume their activity under Green Pass provisions when the vaccine roll-out is at its peak and while a substantial part of the population has been vaccinated or has recovered. When a rather small percentage of the local population has been vaccinated or has recovered, businesses and public places do not have a big enough incentive to resume their activities. On the other hand, when most of the population is immune and communal immunity has been attained, the economic and cultural spheres can resume their activity with no need for any mandatory limitations.

## Equal access to vaccinations and the Green Pass

The differentiation between vaccinated or recovering individuals who are entitled to the Green Pass and individuals who are not is relevant to interrupting infection and therefore does not infringe on the right to equality. Nonetheless, conditioning the admission to businesses and public places on the presentation of a proof of vaccination where accessibility to vaccinations is unequal would indirectly infringe on the right to equality.

Data published by the Israeli Ministry of Health suggests that vaccine compliance is higher among secular Jews and among more affluent sections of the population, which leaves the Arab–Israeli minority and the less well-off behind [19].

The main reasons for this uptake gap were, among others, accessibility difficulties due to the relatively late opening of vaccination centers designated for the Arab–Israeli population, as well as difficulties in accessing reliable information due to the comparatively meagre governmental efforts to fight fake news at an early stage [20].

Given that bringing every person down to the least advantaged position ("levelling down") would not—generally speaking—solve the problem of disadvantage [21], this uptake gap should not serve as a justification for refraining from the implementation of the Green Pass program. However, the implementation of such a program nonetheless underscores the need for making vaccines accessible to vulnerable, and especially poor, populations. Efforts toward making vaccines physically accessible may include individual outreach to those who have yet to be vaccinated, providing access to vaccination centers without the need for setting an appointment in advance (whether online or otherwise), and the use of mobile immunization clinics.

Alongside the right to equal physical access to vaccines, equality also requires health communications that are linguistically and culturally adapted to the needs of various communities. This, in turn, requires an investment in communicating health information in a variety of languages (the relevant languages in Israel would be Hebrew, Arabic, Russian, and Amharic) that would provide a suitable response to "fake news" which has been propagated in these languages on social networks, as well as the recruitment of opinion leaders and medical experts that would be both relevant to as well as respected by the various sub-communities in question.

Insofar as the ethical obligation to reduce the indirect infringement on the right to movement of the unvaccinated is concerned, the Green Pass initiative must be accompanied by such alternatives as rapid COVID-19 testing. Although the results of such tests are less accurate, permitting admission to businesses and public places based on a negative rapid COVD-19 test should be viewed as a calculated and justified risk. The availability of alternatives is especially necessary in cases where the lack of eligibility is not due to choice, but to vaccine contraindications (children under 12 and those who are allergic to one or more vaccine components). In addition, and in a manner akin to the obligation to equal access to vaccines, equal access to COVID-19 tests must also be assured. To this end, rapid COVID-19 tests must be made financially available to all. Governments must act to reduce the price of such tests and to fully fund them in the case of children or other populations who cannot be vaccinated.

## Proportionality in mandating a Green Pass

The Israeli Green Pass is only required as a precondition for admission into places that are considered "luxuries", which serves the objective of reducing the violation of non-Green Pass holders’ rights. The Pass is not required for admission to hospitals, clinics and pharmacies, which is necessary for the realization of the right to health, and public places that combine necessary business with the pursuit of leisure (such as shopping centers), have been opened to the public without restrictions.^3^

However, the legislature has not addressed the legitimacy of requiring a Green Pass from employees as a precondition for their continued employment or hiring. As a result, businesses and public places were allowed to open subject to the presentation of a Green Pass by visitors and customers, while their employees were not required by law to prove that they recovered from COVID-19 or that they were fully vaccinated.

In addressing this issue, the Deputy Attorney General of Israel has stated that employers’ managerial prerogative in deciding to require their employees to present a Green Pass should be balanced against the rights of the working individual, including her or his right to autonomy over her or his body and her or his right to privacy. According to the Deputy Attorney General, it is inappropriate to impose a sweeping prohibition on employers to demand a Green Pass from their employees, but it is also unwarranted to establish a general requirement to present a Green Pass without examining whether such a measure is proportionate. Each case should thus be examined in relation to the concrete circumstances of that specific workplace and every employer should also consider the feasibility of less intrusive alternatives.

This issue has been consequently presented to Israeli Labor Courts in two distinct cases of workers petitioning against their employers' requirement to present a Green Pass as a condition for entering the workplace and claiming that their rights had been unduly violated. In both cases, the courts ruled that the employers' demand was legitimate in light of the circumstances. More specifically, one case involved a teaching assistant who was barred from entering her workplace (a school for children with special needs) as she refused to be vaccinated or to present a negative COVID-19 test. In its decision, the court stated that "…In this case, the petitioner was required to present a negative COVID-19 test once a week… this is a proportionate and reasonable requirement. No less intrusive measure could have been taken against the petitioner given the nature of her work as a teaching assistant, which necessarily requires direct contact with unvaccinated children". The court added that "it is difficult to maintain physical distance and full masking given the children’s nature and age…" [22].

The second case involved a supermarket cashier sent on unpaid leave due to her refusal to get vaccinated or to present a negative COVID-19 test every 3 days. In this case, the court ruled that "…The respondent's decision, made with the consent of the employees' union representatives, is proportionate and reasonable. It was based on a multi-layered [set of] alternatives designed to allow the continued employment of the unvaccinated employees… There is no doubt that the petitioner’s work as a cashier at a supermarket branch involves contact with a large number of customers and with other employees, some of whom may be members of at-risk populations, on a daily basis, a state of affairs which makes the employer's requirement a legitimate one….” The court added that the possibility of giving the applicant alternative tasks which do not involve contact with customers, employees, or suppliers was examined, but found to be non-implementable in practice [23].

Requiring employees to present a Green Pass as a condition for entering their workplaces is, in our opinion, only proportionate if the following conditions are met: First, employees are given the alternative option of undergoing a PCR or a rapid antigen test every few days; Second, the Green Pass is only required where it is necessary for safe business or venue activity. A Green Pass is a legitimate precondition for safe business activity when it is not possible to maintain sufficient physical distance from other employees or customers in the workplace. This is all the more relevant when we relate to work involving at-risk populations: children—who cannot be presently vaccinated, patients, and the elderly, who may suffer from immunodeficiency. The third condition is that measures against an employee shall begin with the least infringing option (such as a change in position or a requirement to work from home) and will only proceed to unpaid leave or dismissal when less intrusive measures are impossible to apply.

With that said, priority should be given to measures taken by the workplace to ensure that the vaccine is accessible to employees. Such measures include providing the opportunity to be vaccinated during work hours, the granting of symbolic benefits to vaccinees, or coordinating the arrival of mobile vaccination units to the workplace. Such measures would promote a safe working environment and express support for the vaccine efforts that would contribute to society as a whole.

## Fairness in Green Pass eligibility criteria

Regardless of the vaccine purchase agreements between the Israeli Ministry of Health and Pfizer Inc. and alongside them, the Israeli Institute for Biological Research (IIBR) has also been developing a vaccine produced by using the genetically-modified virus method. This differs from the mRNA method employed by the Pfizer and Moderna vaccines which have received FDA approval. The development of the IIBR vaccine is currently in Phase 2 of its clinical trials, and aims for about 1,000 volunteer participants, some of whom shall be assigned to a placebo group [24].

This, in turn, has raised the question of whether it is appropriate to issue a Green Pass to individuals recovering from COVID-19 or to individuals who have been vaccinated with the Pfizer vaccine as well as to the volunteers who are participating in the IIBR vaccine trial. Answering this question requires the consideration of two issues: the vaccine developed by the IIBR has yet to prove its effectiveness in preventing COVID-19 morbidity, mortality and infection, and the fact that some of the vaccine trial participants were given a placebo and are definitely unvaccinated. On the other hand, not recognizing the IIBR trial participants as eligible for a Green Pass might be detrimental to the trial due to the withdrawal of participants, which had already taken place a week after the Green Pass program began.

On March 6, 2021, a parliamentary decision granted the Green Pass to trial participants who were given the IIBR vaccine alongside individuals vaccinated with the Pfizer vaccine or individuals who had recovered from COVID-19. Trial participants who were given placebo or low doses of the IIBR vaccine were informed that they are entitled to receive the Pfizer vaccine [25].

We believe this decision is ethically and morally justified. Not granting trial participants the right to a Green Pass would mean negatively rewarding them for stepping up and taking a risk for the sake of the communal goal of vaccine development, and thus endangers that same goal. At the same time, allowing several hundred IIBR trial volunteers to enjoy the advantages of a Green Pass would exert a minuscule impact on public health, and would Acknowledge their efforts and contribution.

## Concluding thoughts

The implementation of a COVID-19 Green Pass program is an imperative that would minimize infringements on the rights to movement, gathering and employment among eligible individuals and allow for the recovery and maintenance of the economic, educational and cultural spheres of activity that are essential for the common good.

Learning from the Israeli experience, we stress the need to integrate ethical values into the Green Pass's framework, while advancing and maintaining its effectiveness in achieving the sought-after communal goals.

Implementing the Green Pass provision while safeguarding equality, proportionality, and fairness may imbue this measure with ethical legitimacy despite involving a potential breach of trust and solidarity.

## Footnotes

1 Trust between policy makers and the public alongside a sense of solidarity have been a cornerstone of Israeli public health actions since the establishment of the state of Israel and have led to high compliance rates with government recommendations in the field of public health. A notable example is the relatively high compliance rates attained with respect to polio vaccine recommendation in 2013, when wild polio was detected in Israeli sewage. Parents were asked to vaccinate their children with OPV (oral polio vaccine) although the children were already protected from the disease. The objective of the additional vaccination was to protect non-immune populations. The relatively high compliance rates in this case were attained without the need for sanctions. During the first two waves of the COVD-19 outbreak in Israel, trust and solidarity led to an impressive degree of public cooperation with the social distancing and lockdown guidelines. This, in turn, resulted in a rapid decline in morbidity. In December 2020, the initiation of Israel’s vaccination campaign without constraints on those who avoid vaccination expressed policymakers’ trust in the public's discretion and prevented unvaccinated individuals from feeling alienated [13].

2 We believe that, unlike the benefits of the Green Pass in resuming routine life activities and safely opening the economy, the use of the Green Pass as a means of pressuring people to be vaccinated is ethically problematic. Vaccine compliance should be promoted by accessibility and outreach efforts. Other measures for promoting vaccine compliance that have also been considered in Israel include monetary incentives to "fully vaccinated individuals", and the provision of the contact details of unvaccinated individuals to local authorities such as to allow local representatives to contact the unvaccinated and promote their compliance. Both interventions were not applied. The first and only direct incentive for vaccine uptake went into force on August 5th 2021. This legal provision stipulated that the unvaccinated population will only be eligible for 75% of state funded sick-days if they are required to quarantine themselves due to a positive COVID-19 test or due to close contact with someone else who had been confirmed as a carrier of COVID-19 [17].

3 In accordance with Israeli regulations, and even though the Green Pass is not required for entry into shopping centers, it is required for entry into leisure businesses located in shopping centers. The lack of enforcement of the requirement to show a Green Pass upon entering these leisure businesses has provoked criticism. The owners of leisure businesses located outdoors have argued for unjust advantages held by leisure businesses located inside shopping centers. We would like to note in this respect that the exemption from the requirement of a Green Pass at critical businesses is legitimate as long as it is limited to those businesses only.
